# Oral dosing for antenatal corticosteroids in the Rhesus macaque

**DOI:** 10.1371/journal.pone.0222817

**Published:** 2019-09-19

**Authors:** Augusto F. Schmidt, Matthew W. Kemp, Mark Milad, Lisa A. Miller, James P. Bridges, Michael W. Clarke, Paranthaman S. Kannan, Alan H. Jobe

**Affiliations:** 1 Department of Neonatology and Pulmonary Biology, Cincinnati Children’s Hospital Medical Center, University of Cincinnati, Cincinnati, Ohio, United States of America; 2 University of Western Australia, Perth, Australia; 3 Milad Pharmaceutical consulting LLC, Plymouth, Michigan, United States of America; 4 California National Primate Research Center, University of California, Davis, Davis, California, United States of America; 5 Metabolomics Australia, Centre for Microscopy, Characterization and Analysis, The University of Western Australia, Perth, WA, Australia; Centre Hospitalier Universitaire Vaudois, FRANCE

## Abstract

Antenatal corticosteroids (ACS) are standard of care for women at risk of preterm delivery, although choice of drug, dose or route have not been systematically evaluated. Further, ACS are infrequently used in low resource environments where most of the mortality from prematurity occurs. We report proof of principle experiments to test betamethasone-phosphate (Beta-P) or dexamethasone-phosphate (Dex-P) given orally in comparison to the clinical treatment with the intramuscular combination drug beta-phosphate plus beta-acetate in a Rhesus Macaque model. First, we performed pharmacokinetic studies in non-pregnant monkeys to compare blood levels of the steroids using oral dosing with Beta-P, Dex-P and an effective maternal intramuscular dose of the beta-acetate component of the clinical treatment. We then evaluated maternal and fetal blood steroid levels with limited fetal sampling under ultrasound guidance in pregnant macaques. We found that oral Beta is more slowly cleared from plasma than oral Dex. The blood levels of both drugs were lower in maternal plasma of pregnant than in non-pregnant macaques. Using the pharmacokinetic data, we treated groups of 6–8 pregnant monkeys with oral Beta-P, oral Dex-P, or the maternal intramuscular clinical treatment and saline controls and measured pressure-volume curves to assess corticosteroid effects on lung maturation at 5d. Oral Beta-P improved the pressure-volume curves similarly to the clinical treatment. Oral Dex-P gave more variable and nonsignificant responses. We then compared gene expression in the fetal lung, liver and hippocampus between oral Beta-P and the clinical treatment by RNA-sequencing. The transcriptomes were largely similar with small gene expression differences in the lung and liver, and no differences in the hippocampus between the groups. As proof of principle, ACS therapy can be effective using inexpensive and widely available oral drugs. Clinical dosing strategies must carefully consider the pharmacokinetics of oral Beta-P or Dex-P to minimize fetal exposure while achieving the desired treatment responses.

## Introduction

Although Standard of Care for women at risk of preterm delivery in high income countries [1, 2], antenatal corticosteroids (ACS) are inconsistently used in low resource environments despite endorsement of the World Health Organization [3]. ACS were considered to be the number one intervention to decrease mortality from prematurity in low resource environments in a 2015 World Health Organization report [4]. A recent analysis emphasizes that ACS alone may have only a modest impact on mortality without other improvements in diagnostic, obstetric and newborn care [5]. And there are potential risks of ACS in low resource environments such as hypoglycemia that may be unrecognized an untreated [6]. The largest trial in low resource environments demonstrated no benefit for low birth weight infants and increased perinatal mortality for all treated pregnancies [7]. A general concern about ACS is that the choice of drug and treatment schedule has not been optimized despite indications that current dosing strategies may expose the fetus to higher doses of these potent corticosteroids than is necessary for the desired maturational effects [8, 9]. The accepted ACS treatments are maternal Intramuscular with either a 1 to 1 mixture of betamethasone phosphate and betamethasone acetate (Beta-P + Beta-Ac) given as two 12 mg doses 24 hour apart or four doses maternal intramuscular of 6 mg dexamethasone phosphate (Dex-P) given at 12 hour intervals [4, 10]. We demonstrated that a single dose of Beta-Ac alone that exposed the fetus to low Beta levels was equivalent to the clinical treatment for lung maturational responses in fetal sheep and monkeys [11, 12]. Maternal infusions of Beta-P to achieve low constant fetal exposures to Beta also cause lung maturation in fetal sheep [13]. However, Beta-Ac is not available as a single component drug and maternal infusions are impractical, particularly in low resource environments. As corticosteroid toxicity is proportionate to dose even for short-term exposures [14], our long-term goal is to develop a lower dose and presumably safer treatment option for ACS. We recently reported that oral corticosteroids were effective for ACS in a sheep model [15]. We have extended these observations to the non-human primate by evaluating the pharmacokinetics and lung maturational effects of oral Dex-P and Beta-P as inexpensive and readily available alternatives for ACS. The study also includes mRNA sequencing of fetal lung, hippocampus and liver to evaluate both lung and systemic effects of oral dosing of ACS. These primate studies are essential proof of concept observations that can help justify randomized controlled trials of new ACS treatment options for worldwide use.

## Methods

### Overview of animal studies

Rhesus macaques were studied in the California National Primate Research Center at the University of California Davis with approved procedures and protocols (Protocol #20333). For ultrasonography animals received intramuscular injections of Ketamine or Telazol at appropriate dosing. For hysterotomy, animals receive analgesia followed by inhalation anesthesia using isoflurane performed by a CNPRC veterinarian and anesthesia technician. Procedures were clustered to minimize pain, distress and reduce animal manipulations. After delivery Rhesus macaques fetuses were euthanized with an overdose of pentobarbital (60mg/kg) prior to necropsy. Rhesus mothers that were not euthanized after delivery received 0.15mg/kg of oxymorphone 3 times a day for 3 days for postoperative analgesia. The CNPRC staff carefully observed the animals post anesthesia until awake and active. Animals were monitored daily for food intake, fecal output, hydration and activity. Discomfort was scored daily for 3 days and sutures assessed daily for 7 days post-operative. Animals were monitored for infection, suture reaction or dehiscence. Animals were treated as considered appropriate by the CNPRC veterinary staff. In case the attending veterinarian at the CNPRC deemed euthanasia of the mother necessary for clinical reasons, it was performed with an overdose of IV pentobarbital (60mg/kg).

Measurements of Beta and Dex were made in non-pregnant reproductive age animals and selectively in gestationally dated pregnant animals. Other gestationally dated animals were treated with the maternal, oral or intramuscular corticosteroids or with a saline placebo and the fetuses were delivered in cull by cesarean section 5 days after the initiation of treatments. The descriptions of the treatment groups, gestational ages, birth weights and fetal sex of the animals are in **Table 1**. The Beta-P + Beta-Ac (Celestone®, Merck, Sharp and Dohme, Kenilworth, NJ) and Beta-Ac (a gift of this component of the clinical product from Merck, Sharp and Dohme) were given by intramuscular injections. The oral Beta-P (Focus Pharmaceuticals Ltd., London, UK) and Dex-P (Fresenius Kabi USA LLC, Lake Zurich, IL) were given in bananas to monkeys habituated to receiving small treats. The drugs were mixed into Kool Aid and rice cereal that were embedded in the middle of 1/4 of a banana.

**Table 1:** Animal groups and treatments.

Treatment Group	(N)	Purpose	Dosing	Treatment to delivery interval (days)	GA at delivery (days)	Fetal Weight (g)	Fetal Sex (M/F)
Controls	6	Negative Control	Saline	5	132 ± 2	320 ± 37	4/2
Beta-Ac + Beta-P Maternal IM	6	Clinical dosing–Positive control	0.25 mg/kg x 2–0 and 24 hr.	5	133 ± 1	330 ± 46	4/2
Beta-Ac–Maternal IM	3	Adult non-pregnant blood levels	0.125 mg/kg x 1	—	—	—	—
	4	Maternal and fetal blood levels	0.125 mg/kg x 2–0 and 24 hr.	5	130 ± 3	291 ± 19	2/2
Dex-P—Oral	3	Low dose Adult non-pregnant blood levels	0.04 mg/kg x1	—	—	—	—
	3	High dose Adult non-pregnant blood levels	0.15 mg/kg x 1	—	—	—	—
	4	Maternal and fetal blood levels	0.15 mg/kg x2–0 and 24 hr.	5	130 ± 3	320 ± 26	1/3
	5	Efficacy—Low dose	0.04 mg/kg x4–6 hr interval	5	130 ± 2	387 ± 38	4/2
	8	Efficacy–blood levels	0.15 mg/kg x3–12 hr interval	5	131 ± 1	131 ± 48	6/2
Beta-P—Oral	3	Adult non-pregnant blood levels	0.15 mg/kg x 1	—	—	—	—
	7	Efficacy	0.15 mg/kg x 3–12 hr interval	5	130 ± 2	300 ± 34	3/4

The groups of non-pregnant animals used for pharmacokinetic studies had been acculturated to blood draws without restraint or anesthesia. Limited paired maternal and fetal blood samples from animals sedated with Ketamine were collected using ultrasound to guide the collection of a small volume of blood from umbilical cord and venous blood from the dam. Fetuses at delivery had blood drawn from the cord vessels just prior to euthanasia with pentobarbital given IV. The fetal chest was opened, the trachea cannulated, and a pressure-volume curve was measured using a syringe and manometer to inflate the lung to 40 cm H_2_O pressure with air and then to deflate the lung with recording of pressure and volume [16]. The lungs were weighed, and the left lung was used for four pooled alveolar washes with saline. The right lung lower lobes were sampled for cryopreservation and the right upper lobe was inflation fixed at 30 cm H_2_O pressure with 10% formalin.

### Measurements on maternal and fetal blood and alveolar wash

We measured complete blood counts, CD4, CD8 and B-cells by FACS, and plasma glucose on selected samples. Cortisol was measured using an ELISA kit (EA65, Oxford Biomedical Research, Rochester Hills, MI). Plasma was extracted with deuterated Beta or Dex added to estimate recovery efficacy and analyzed by mass spectrometry as before [17]. Saturated phosphatidylcholine‎ (SAT-PC) in alveolar wash was measured by phosphorous assay following lipid extraction and treatment of the lipid extracts with osmium tetroxide [18]. Surfactant protein SP-D was measured in alveolar wash by ELISA using a SPD detection kit (cat# RD194059101, Biovendor, Ashville NC).

### mRNA measurements

We extracted total RNA from frozen lungs, hippocampus and liver using RNeasy Universal Mini kits (Qiagen, Valencia, CA). Specific sequences for Rhesus were used to measure mRNA in lung for the surfactant proteins SP-A, B, C, D and other maturational mRNA marker genes (ABCA3, LAMP3, LPCAT1 and SCNN1G) by PCR.

Quality and integrity of total RNA from fetal lung, liver and hippocampus were verified for RNA sequencing using the Agilent 2100 Bioanalyzer (Agilent, Agilent Technologies, Santa Clara, CA). The Cincinnati Children’s Hospital Medical Center DNA Sequencing and Genotyping Core performed sequencing with a read depth of 20–30 million reads per sample for 75 bp with paired-end reads. The raw sequence reads in FASTQ format were aligned to the Rhesus (*Macaca mulatta*) genome build MMUL1.0 using STAR [19]. Differential expression analyses comparing oral Beta-P and the clinical treatment was performed using DESeq2 [20]. Genes were considered differentially expressed based in their fold-change relative to control (= or >1.5), p-value (<0.05) and q-value (<0.1).

### Functional enrichment and pathway analysis

Lists of differentially expressed genes were used for functional enrichment analysis using the g:Profiler web server filtered to exclude electronic GO annotations and false discovery rate adjustment by the Benjamin-Hochberg method [21]. Enrichment p-values are presented as log p-values with negative values representing terms associated with suppressed genes and positive values representing terms associated with induced genes.

### Statistics

Values are given as means ± SD with 2 group comparisons using T-tests or multiple comparisons using ANOVA as appropriate.

## Results

### Pharmacokinetics

**Fig 1:**
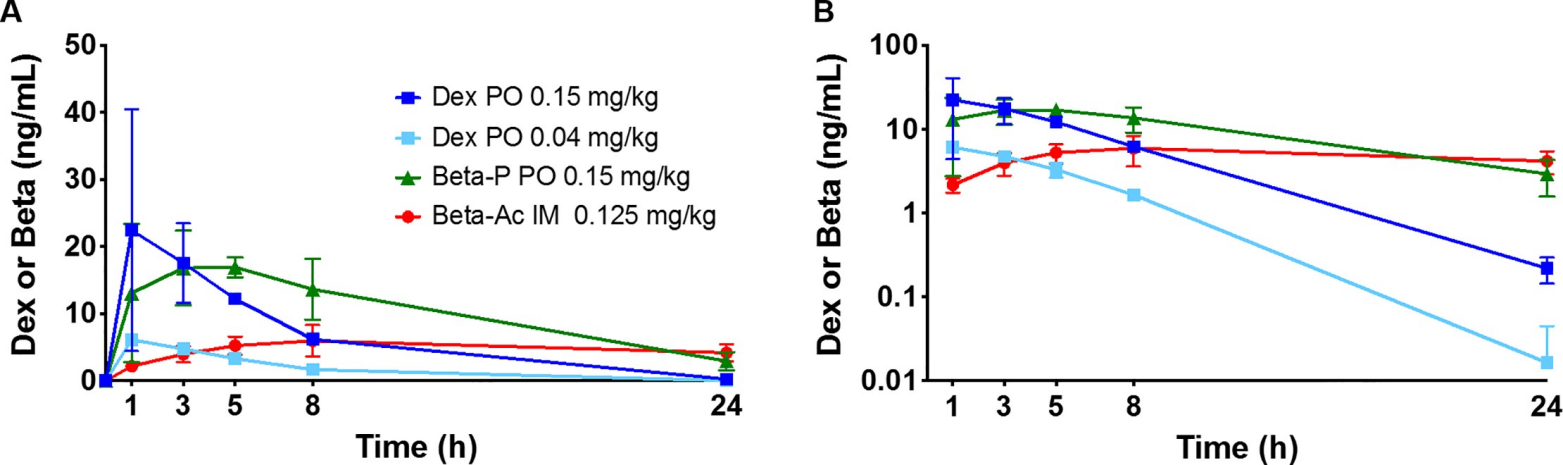
Pharmacokinetics of corticosteroids in non-pregnant reproductive age Rhesus macaque given on a linear scale (A) and log scale of concentration (B). The separate groups of 3 animals were treated with 0.15 mg/kg dexamethasone phosphate orally, 0.04 mg/kg dexamethasone phosphate orally, 0.15 mg/kg betamethasone phosphate orally, or 0.125 mg/kg betamethasone acetate intramuscular. Blood samples were collected over 24 hr. for analysis of betamethasone or dexamethasone in plasma and reported as ng/mL.

Our measurements in primates were guided by pharmacokinetic and lung maturation measurements that we have made in a fetal sheep model using Beta-P+Beta-Ac and Beta-Ac given maternal intramuscular, Beta-P given by maternal infusions and oral treatments with Beta-P and Dex-P [11, 13, 15]. In the fetal sheep, plasma Beta levels in the range of 1 to 4 ng/mL caused lung maturation if maintained for at least 24 hrs. [13, 22]. A dose of 0.125 mg/kg Beta-Ac also caused lung maturation in the monkey and a lower dose was not effective [12]. We used the effective dose of 0.125 mg/kg of the Beta-Ac component of the clinical combination drug to determine maternal blood levels that should be effective for lung maturation. Fetal plasma Beta levels from Beta-Ac were 2.2 ± 0.4 ng/mL at 1hr. with the highest value of 5.9 ± 2.4 ng/mL at 8hr. and with minimal clearance to 24 hr. in non-pregnant monkeys (**Fig 1, Table 2**). In pregnant humans, oral Dex-P has a bioavailability of about 75% of intramuscular Dex-P in two reports [23, 24]. Therefore, we increased the oral doses of Dex-P and Beta-P to 0.15 mg/kg for equivalence with the Beta-Ac results. Oral dosing with 0.15 mg/kg Dex-P or Beta-P yielded comparable blood levels in nonpregnant monkeys of 17.4 ± 5.9 and 16.8 ± 5.6 ng/mL at 3 hrs., values that were about 4-fold higher than for Beta-Ac. Both drugs had rapid oral absorption that was similar. The subsequent half-life values of 3.3 hrs. for Dex and 7.4 hrs. for Beta were quite different with maternal blood levels at 24 hrs. of 2.9±1.4 ng/mL for Beta and 0.22 to 0.07 ng/mL for Dex. Therefore, for considerations of oral dosing, either drug achieved higher initial plasma levels more rapidly than Beta-Ac, but the slower clearance of Beta would favor Beta-P for oral dosing as re-treatment would be needed less frequently to keep the steroid level above 1 ng/mL. The plasma levels of a low dose of 0.04 mg/kg oral Dex-P were about 25% of the high dose, indicating proportionate exposures based on dose. This experiment provided us with information to estimate fetal plasma levels for testing oral maternal treatments in pregnant monkeys.

**Table 2:** Pharmacokinetic measurements from non-pregnant Rhesus.

	Beta-Ac 0.125 mg/kg IM	Dex-P 0.15 mg/kg PO	Beta-P 0.15 mg/kg PO	Dex-P 0.04 mg/kg PO
C_max_ (ng/mL)	6.1 ± 2.2	26.2 ± 14.8	20.3 ± 2.3	6.1 ± 0.7
AUC _24_	113 ± 38	134 ± 32	226 ± 23	32 ± 6.8
t_1/2_ (hrs.)	N/A	3.3	2.5	7.4

N/A: The half-life (t1/2) after intramuscular Beta-Ac could not be estimated.

**Fig 2:**
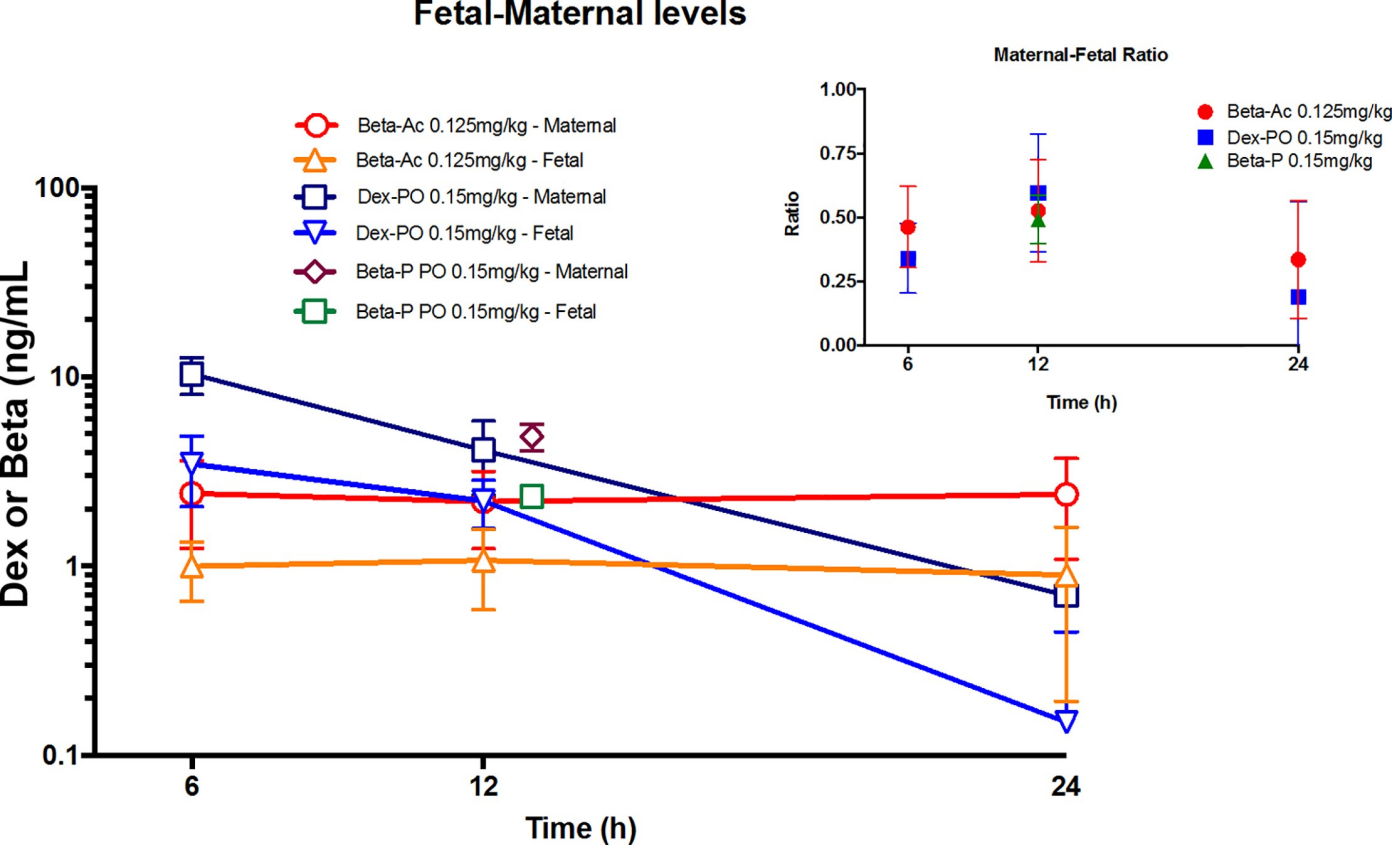
Pharmacokinetics of corticosteroids in maternal and fetal plasma. Groups of 4 pregnant monkeys were treated with maternal intramuscular 0.125 mg/kg betamethasone-acetate, maternal oral 0.15 mg/kg dexamethasone-phosphate or maternal oral 0.15 mg/kg betamethasone-phosphate (Beta-P). Paired maternal and fetal blood samples were collected 6, 12 and 24 hr. after the intramuscular betamethasone-acetate and oral dexamethasone-phosphate treatments and only at 12 hr. for the betamethasone-acetate. Plasma levels are reported as ng/mL. The insert gives the ratios for fetal to maternal drug ratios.

We then did a minimal sampling protocol of paired maternal blood and ultrasound guided fetal blood collections at 6 hr, 12 hr, and 24 hr after treatment without complications or loss of a fetus. We again used maternal intramuscular 0.125 mg/kg Beta-Ac as the known effective dose and the same 0.15 mg/kg oral Dex-P (**Fig 2**). Beta levels from Beta-Ac were essentially constant over 24 hrs. in the maternal plasma at about 2.5 ng/mL, a value lower than the value of about 5 ng/mL in the non-pregnant animals. Fetal blood levels also were stable over 24 hrs. at about 1 ng/mL with an average fetal to maternal ratio of 0.42. The oral Dex-P yielded a higher maternal plasma value of 10.4 ng/mL at 6 hr. but by 24 hr. the value was 0.7±0.4 ng/mL with a half-life of 4.8 hr. Maternal blood Dex levels were similar to the levels for the nonpregnant monkeys. The t_1/2_ for Dex in fetal blood was 4.3 hrs. from 6 to 24 hrs. with a maternal half-life of 4.8 hr. In 3 animals we also had paired maternal and fetal blood sampling at 12 hr. only following a maternal dose of 0.15 mg/kg Beta-P (**Fig 2**). The maternal values were comparable to the oral Dex-P levels in the non-pregnant monkey with a fetal to maternal ratio for Beta of 0.49. Given the slower clearance of Beta from the maternal circulation, estimated fetal levels at 24 hr. should be about 1.5 ng/mL.

### Lung pharmacodynamic effects

**Fig 3:**
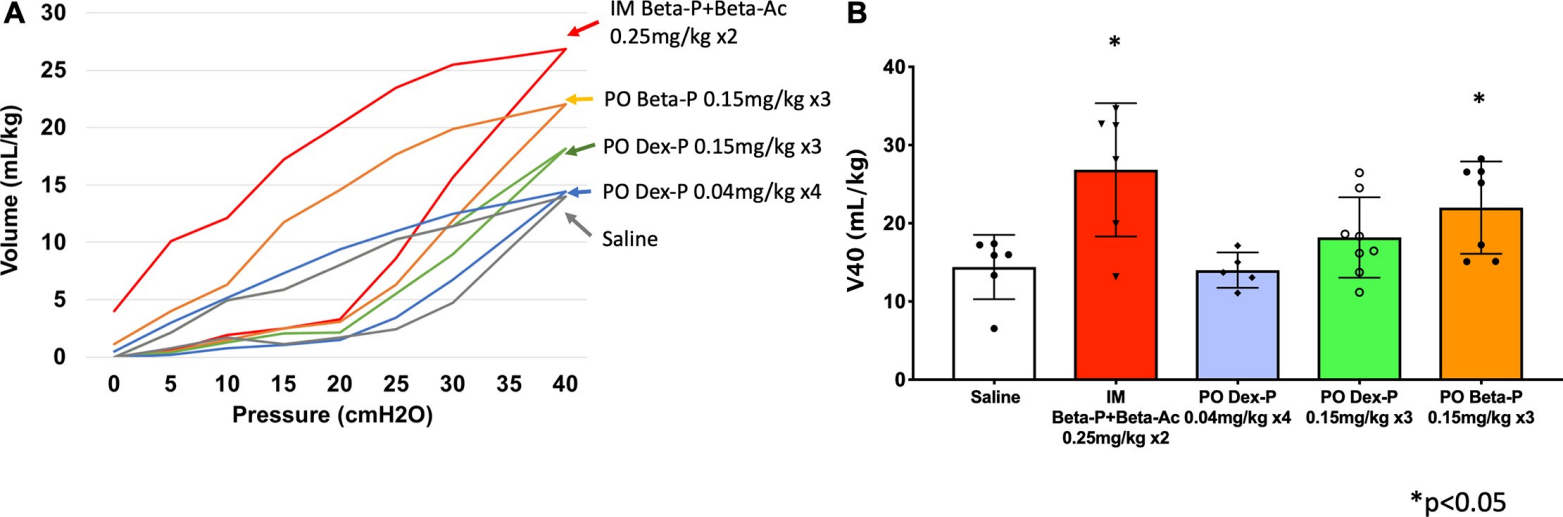
A. Pressure-volume curves and B. maximal lung gas volumes measured at 40 cmH_2_O pressure for groups of 6 to 8 fetuses 5 days after the maternal treatments as indicated. The clinical treatment and oral Beta-P group were significantly different from saline controls (p<0.05).

We then tested oral Beta-P and oral Dex-P in comparison to saline controls and the positive control with two intramuscular doses of 0.25 mg/kg Beta-Ac + Beta-P given 24 hrs. apart. We measured pressure-volume curves 5 days after the initial dose to assess static lung compliance by the gas volumes in the lung at 40 cm H_2_O peak pressure (V40), (**Fig 3)**. Based on the low fetal plasma levels at 24 hr. for oral Beta-P and Dex-P (**Fig 2**) we tested efficacy with 3 oral doses of 0.15 mg/kg given at 12 hr. intervals. Beta-P given as three 0.15 mg/kg oral doses separated by 12 hrs. yielded pressure-volume curves that were significantly different from controls and were similar to the mean curve for the clinical dosing. The qualitative V40 and deflation stability values were not significantly different relative to the saline control for the 8 animals treated with Dex-P. Of note, 2 of the 8 animals had increased lung gas volumes comparable to the mean value for the clinical treatment with 2 intramuscular doses of 0.25 mg/kg Beta-P + Beta-Ac. With low dose 0.04 mg/kg oral Dex-P treatment given as 4 doses at 6 hr. intervals the mean pressure volume curves demonstrated no effect. We also gave 2 doses of 0.125 mg/kg of Dex-P separated by 24 hr. to three animals that were used for maternal to fetal drug transfer measurements. The V40 values were 18 ± 5 ml/kg, which was not different than for the saline control curves of 16 ± 2 ml/kg at 5 days. Sat-PC in the alveolar washes qualitatively increased in the 3 steroid treated groups but was not significant for any group because of highly variable values (**Table 3**). Surfactant protein D (SP-D) also was measured in the alveolar wash and was not different across groups. Selected mRNA measurements associated with lung maturation were measured by PCR, and there were no changes in these mRNA values relative to the controls at 5 days.

**Table 3:** Measurements at 5 days after treatment.

	Control Saline	Clinical—0.25 mg/kg Beta-P + Beta-Ac (0 and 24 hrs.)	Oral Dex-P 0.15 mg/kg (0, 12, 24 hrs.)	Oral Beta-P 0.15 mg/kg (0, 12, 24 hrs.)
Fetal weight (g)	320 ± 37	330 ± 46	331 ± 48	300 ± 34
Lung weight / fetal weight (g/kg)	20 ± 10	26 ± 2	24 ± 3	27 ± 4
Thymus weight / fetal weight (g/kg)	3.4 ± 0.8	2.6 ± 0.5	2.7 ± 0.3	2.5 ± 0.7
***In Bronchoalveolar Lavag***e				
Sat Phosphatidylcholine (μm/kg)	0.12 ± 0.05	0.68 ± 0.89	0.13 ± 0.13	0.13 ± 0.13
Surfactant Protein-D (ng/mL)	457 ± 216	776 ± 438	733 ± 580	733 ± 580
***In maternal blood***				
CD-4 lymphocytes % total	8.7 ± 3.7	8.4 ± 3.9	6.1 ± 4.0	4.5 ± 8.3
CD-8 lymphocytes % total	7.1 ± 3.4	8.7 ± 3.3	7.6 ± 2.7	4.8 ± 2.7
B cells % total	3.8 ± 2.4	3.9 ± 1.7	3.1 ± 0.8	2.9 ± 1.4
Cortisol (μg/dL)	17.1 ± 0.3	10.8 ± 4.5*	18.6 ± 10	15.9 ± 7.8
***In fetal blool***				
Cortisol (μg/dL)	2.9 ± 0.8	2.0 ± 0.5	3.2 ± 0.8	2.4 ± 0.6
***In mRNA fetal lung tissue***				
SP-A	1 ± 0.35	1.36 ± 0.57	1.35 ± 0.68	1.47 ± 1.10
SP-B	1 ± 0.28	1.31 ± 0.47	0.97 ± 0.29	0.94 ± 0.38
SP-C	1 ± 0.48	1.22 ± 0.54	0.72 ± 0.24	0.65 ± 0.27
SP-D	1 ± 0.33	1.16 ± 0.39	1.01 ± 0.27	0.87 ± 0.34
ABCA3	1 ± 0.17	1.23 ± 0.30	1.06 ± 0.37	1.00 ± 0.43
LAMP3	1 ± 0.27	1.10 ± 0.67	0.72 ± 0.15	0.67 ± 0.33
LPCAT1	1 ± 0.18	1.26 ± 0.36	1.18 ± 0.41	0.92 ± 0.41
SCNN1G	1 ± 0.28	1.15 ± 0.30	1.15 ± 0.32	0.94 ± 0.32

*p<0.05 compared to control

### Other effects of the treatments

Only the clinical treatment caused suppression of maternal cortisol at 5 days (Table 3). The fetal weights (**Table 1**), lung weights and thymus weights (**Table** 3) were not different across the 5 day treatment groups. The dam white blood cell values, lymphocyte classes and glucose values also were not different at 5 days.

### RNA-sequencing

**Fig 4:**
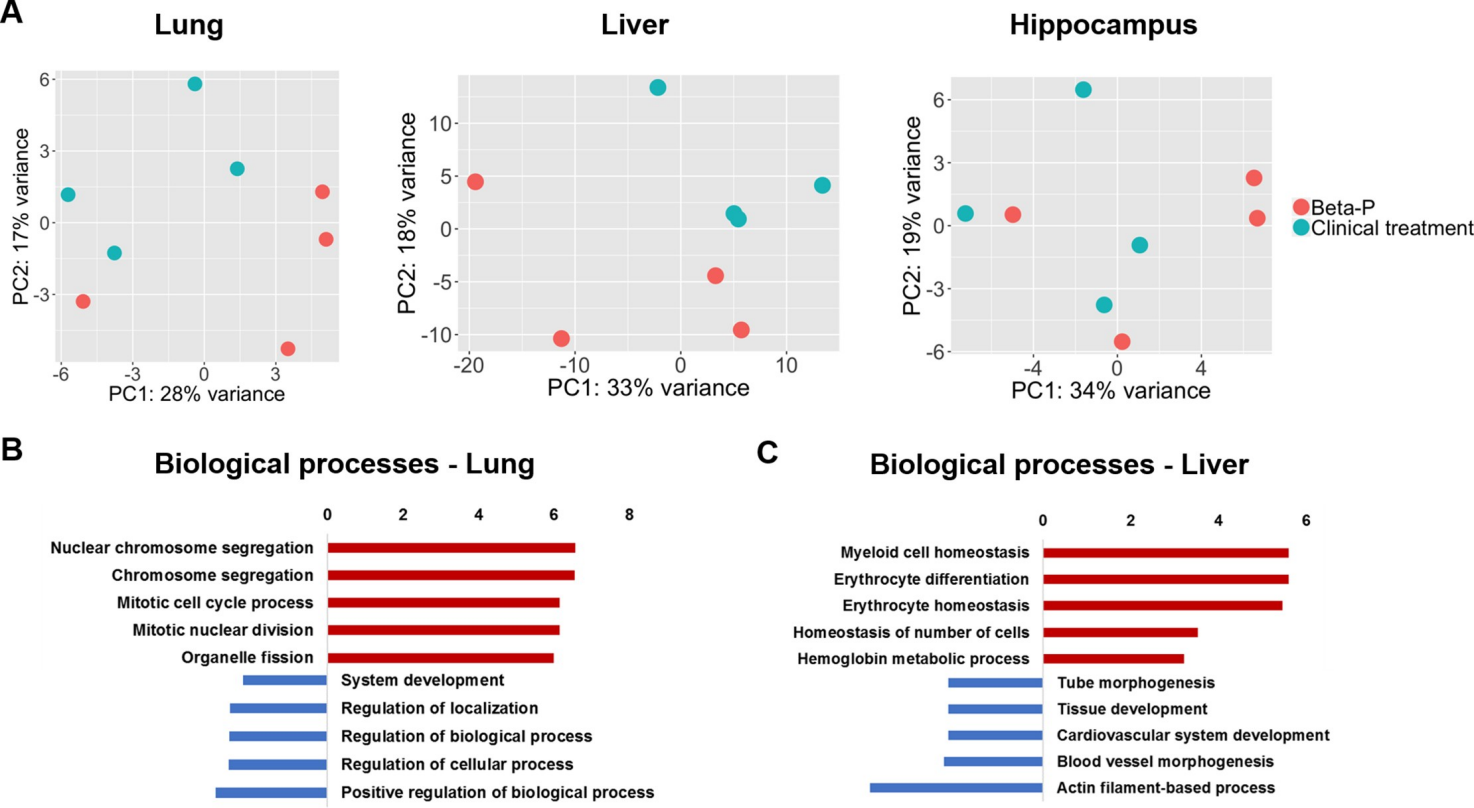
A. Principal component analysis of transcriptomic profiles of fetuses treated with the clinical treatment or oral Beta-P (n = 4 animals/group). B. Biological processes associated with differentially regulated genes in the lung. C. Biological processes associated with differentially regulated genes in the liver.

**Fig 5:**
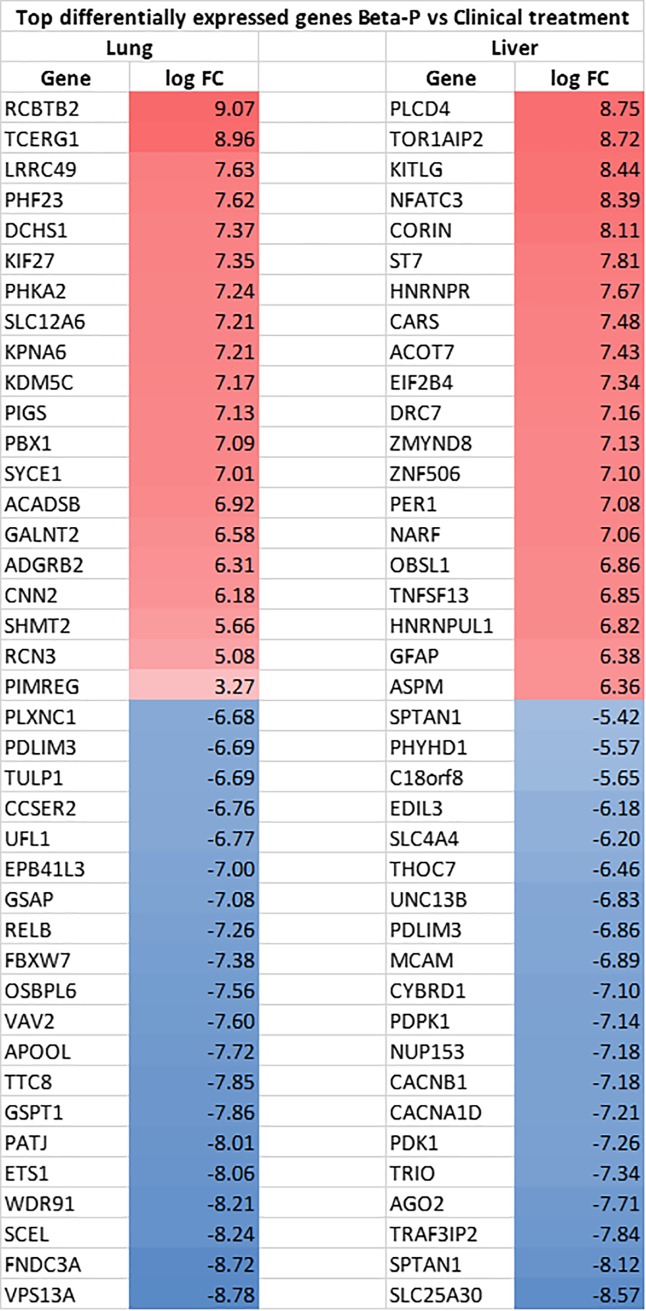
Top differentially expressed genes by RNA-sequencing in the lung and liver of animals treated with Oral Beta-P compared to the clinical treatment. Values are expressed as log fold change (logFC).

In order to identify differential pulmonary and systemic effects of the oral Beta-P treatment compared to the 2-dose clinical treatment we compared the transcriptome profiles of the lungs, liver and hippocampus of 4 animals in each group. We previously reported that the clinical drug has a large impact in the lung and hippocampus transcriptome at an early timepoint of 4h with decreased effects at 5 days [12]. Here we compared the two treatments to each other to explore differential effects of oral dosing and the intramuscular clinical treatment. Principal component analysis showed similarity between groups with little separation between the samples in the two groups (**Fig 4A**). There were no differentially expressed genes in the hippocampus between the two treatments. In the lung, relative to the clinical treatment, oral Beta-P induced 118 and suppressed 236 genes in the lung. The induced genes in the lung were associated with biological processes of cellular division while suppressed genes were associated with developmental processes and differentiation (**Fig 4B**). In the liver, oral Beta-P induced 145 and suppressed 156 genes relative to the clinical treatment. Induced genes were associated with myeloid cell differentiation and suppressed genes were associated with developmental processes, including morphogenesis and angiogenesis (**Fig 4C**). The top differentially expressed genes are listed in **Fig 5**. There were no significant differences in the transcriptome of the hippocampus between groups.

## Discussion

The standard of care for ACS is by repeated maternal intramuscular injections, using several fluorinated corticosteroid preparations that cross the placenta to the fetus [10, 25]. However, the pharmacokinetics to establish optimal drug choice, dose and treatment were never evaluated [9]. Other routes of treatment have been reported but have been minimally studied [10]. Fetal intramuscular steroids were considered for human use to selectively target the fetus, but fetal intramuscular corticosteroids were not as effective as maternal ACS in sheep models [26]. Intravascular ACS using Dex-P also has been reported but will cause very high maternal plasma corticosteroid levels. There are no experimental reports of oral ACS in animals, and the clinical experience with oral ACS is limited to one trial and case reports. Egerman and colleagues reported in 1997 the pharmacokinetics of 6 mg intramuscular Dex-P and 8 mg PO Dex-P in third trimester pregnant women [27]. Peak plasma levels for maternal oral Dex were about 65% of intramuscular levels with peak concentrations at 30 min. for intramuscular and 2 hr. for PO treatments. Plasma half-life values and bioactivity were similar. They then reported a clinical trial in 1998 for women at risk for preterm delivery to receive 4 doses of 6 mg Beta-P intramuscular or 4 doses of 8 mg Beta-P PO, repeated at 12 hr. intervals [23]. This study was stopped at 39% enrollment for more neonatal sepsis and intraventricular hemorrhage in the oral treatment group. There was no explanation for the increase in adverse outcomes with the oral treatment, but the adverse outcomes were lower than expected in the intramuscular treated patients. This trial is a cautionary note indicating that careful monitoring is essential for future trials with oral ACS.

For low resource environments where drug stability, safety and cost are of paramount concern, oral ACS may have many advantages. Beta-P and Dex-P are available as pills or solutions worldwide and are used as oral preparations for multiple indications [14]. This exploratory study was designed as an initial proof of principle of oral ACS using a primate model. We evaluated the pharmacokinetics of oral Beta-P and Dex-P as they are the two fluorinated corticosteroid drugs routinely used for ACS. Our comparison was to the slowly solubilized intramuscular Beta-Ac to calibrate oral dosing against an effective treatment with low fetal exposures that we had characterized in sheep and primate models [11, 12, 15]. In humans, oral Dex-P is rapidly absorbed with a time to peak concentration that is similar or slightly longer than for intramuscular Dex-P and with a clearance half-life of about 4 hrs. in adult men [28]. Peak blood levels of Dex after oral Dex-P are about 70% of the levels following intramuscular Dex-P with clearance half-life values of about 4 hrs. with similar bioavailability for the oral treatment ranging from 70–100% [24, 27]. There is a lack of information comparing oral and intramuscular Beta-P in humans. There also is no information for efficacy of lower doses of these drugs for ACS.

In the non-pregnant female monkey, oral absorption of Dex and Beta were equivalent, but the clearance rate was substantially slower for Beta than for Dex. In the pregnant animals our limited measurements demonstrated stable and low maternal plasma levels from 0.125 mg/kg Beta-Ac of about 2 ng/mL Beta and fetal plasma levels of about 1 ng/mL for the 3 measurements at 6, 12 and 24 hrs. The fetal to maternal Beta ratio was 0.4 in cord blood, which was similar to values reported for humans [24, 29]. The oral dose of 0.15 mg/kg Dex-P demonstrated a maternal half-life of 4.8 hr. and a fetal half-life of 4.3 hr. from 6 to 24 hrs. resulting in a fetal Dex level of < 1 ng/ml by 24 hrs.

For evaluation of treatment effects, we used the doses for which we had the pharmacokinetic measurements in comparison to the much higher clinical treatment with 0.25 mg/kg Beta-P + Beta-Ac given as 2 doses separated by 24 hrs. We assume the clinical treatment causes a maximal ACS response [8]. The lung gas volume responses were measured at 5 days after treatment initiation to be within the interval of 24 hr. to 7 days for clinical benefit [4, 30]. The only physiological measurement available to us with the monkey was the pressure-volume curve. This measurement has reliably predicted dynamic compliance and gas exchange maturational signals in sheep models, including for oral treatment [15]. Statistically, the 0.15 mg/kg oral Beta-P given as 3 doses at 12 hr. intervals was different from the controls and not different from the clinical treatment. The V40 values were more scattered with the 3 dose 0.15 mg/kg Dex-P treatment with 2 of 8 with high V40 values but 6 values were not different from control. The 4 doses of 0.04 mg/kg oral Dex group were clearly not effective. Based on our demonstration that the sustained fetal Beta levels achieved with Beta-Ac are sufficient for lung maturation in sheep and monkeys [11, 12], and the oral fetal Beta level measured at 12 hr. in 2 animals of 2.35 ± 0.05, lung maturation should have occurred (**Fig 2**). The fetal Dex level of 2.2 ± 0.6 measured for the oral Dex dose of 0.15 mg/kg also should have been effective, but variability between animals and the possibility that Dex and Beta are not equivalent by potency may explain the lack of effect. In humans, the 2-dose ACS treatment decreased respiratory distress syndrome by only 40%, and ACS treatments in animal models consistently have variable responses with animals that do not have lung maturation responses [1, 11]. In this study, 2 of the 6 animals given the high 2 dose clinical treatment were in the range of the control animals (**Fig 3**). Variable responses and the limitation of animal numbers when using primates makes these experiments of low sensitivity.

In a previous study with this same model with primates, single doses of intramuscular 0.25 mg/kg Beta-P+Beta-Ac and 0.125 mg/kg Beta-Ac increased the amount of Sat PC in the alveolar lavage at 5 days as an indicator of increased surfactant by ACS [12]. In this experiment, two dose of 0.25 mg/kg Beta-P+Beta-Ac or oral dosing with Beta-P or Dex-P did not significantly increase SAT-PC. The estimates of surfactant pool sizes are remarkably low—0.25 mg/kg for controls and 0.66 mg/kg for the two dose 0.25mg/kg Beta-P+Beta-Ac. Similarly, surfactant protein D did not increase, and we did not have assays for the other surfactant proteins. The panel of mRNAs as indicators of maturation also were not increased at 5 days, a result we anticipated. In sheep models, increases in mRNA occur quickly after fetal exposure to ACS or inflammation, but the mRNA values return to control values within a few days [31]. The same phenomenon has been described for explants from early gestation human lung [32].

We showed in the primate model that eliminating unnecessary peak fetal exposure to antenatal corticosteroids decreases potentially toxic effects identified in the fetal hippocampus by transcriptome analysis [12]. Despite the higher dose that was administered orally, the differences in the transcriptome of the liver and lung after oral treatment with Beta-P compared to the clinical treatment were small and do not indicate the potential for increased toxicity. In the lung Beta-Ac had increased expression of genes associated with cellular proliferation. Suppression of cellular proliferation is a known effect of corticosteroids that we had previously observed with a single dose of the clinical drug and here we observe with 2 doses of the clinical drug relative to the oral Beta-Ac. The suppression of genes associated with development likely reflect effects of corticosteroids on differentiation and maturation pathways but are of unclear significance as the genes affected are not important mediators of fetal lung maturation and the functional maturation measured by lung compliance was similar between the two treatments. More importantly, there were no differences in the hippocampus transcriptome showing that the oral dose does not expose the fetus to additional potential risks to neurodevelopment compared to the clinical treatment.

The experiments have limitations inherent to studies in primates. Our assessments of lung responses in small experimental group sizes compromised the sensitivity of the studies. In contrast to similar studies in sheep models, we did not attempt to ventilate the preterm monkeys after delivery to evaluate dynamic lung mechanics and gas exchange [12]. The pregnant monkeys had lower plasma corticosteroid levels than did the non-pregnant monkeys, which needs to be further evaluated in the human. The clearance kinetics for Beta and Dex were quite different. Any future clinical studies of oral dosing of ACS need to consider the pharmacokinetics of the drug chosen for evaluation.

In conclusion, oral ACS treatments are feasible. Dosing can be adjusted based on pharmacokinetics to achieve fetal exposures that avoid high and unnecessary fetal plasma corticosteroid levels, as demonstrated for intramuscular treatments [33]. Reliable oral dosing may depend on variable such as recent food consumption, maternal weight which will need to be explored clinically. This report together with our other recent studies in sheep and monkey models establish the rational for developing knowledge based approaches to new ANS treatment strategies [12, 15].
